# Antibacterial and Antioxidant Properties of Extracts of *Yucca Baccata*, a Plant of Northwestern Mexico, against Pathogenic Bacteria

**DOI:** 10.1155/2022/9158836

**Published:** 2022-10-26

**Authors:** Gloria-Guadalupe Morales-Figueroa, Gabriel Darío Pereo-Vega, Manuel Everardo Reyna-Murrieta, Rosalva Pérez-Morales, Marco Antonio López-Mata, José Jesús Sánchez-Escalante, Melvin R. Tapia-Rodriguez, J. Fernando Ayala-Zavala, Josué Juárez, Luis Quihui-Cota

**Affiliations:** ^1^Departamento de Nutrición Pública y Salud, Coordinación de Nutrición, Centro de Investigación en Alimentación y Desarrollo, A.C. Carretera Gustavo Enrique Astiazarán Rosas, No. 46. Col. La Victoria, 83304 Hermosillo, Sonora, Mexico; ^2^Coordinación de Ciencias de los Alimentos, Centro de Investigación en Alimentación y Desarrollo, A.C. Carretera Gustavo Enrique Astiazarán Rosas, No. 46 Col. La Victoria, 83304 Hermosillo, Sonora, Mexico; ^3^Departamento de Ciencias de la Salud, Universidad de Sonora, Campus Cajeme, Blvd. Bordo Nuevo s/n, Antiguo Ejido Providencia, Apdo, 85040 Ciudad Obregón, Sonora, Mexico; ^4^Herbario USON. Departamento de Investigaciones Científicas y Tecnológicas, Universidad de Sonora, Unidad Centro, Apdo, 83000 Hermosillo, Sonora, Mexico; ^5^Departamento de Biotecnología y Ciencias Alimentarias, Instituto Tecnológico de Sonora, Calle 5 de febrero No. 818 sur, Ciudad Obregón, Sonora 85000, Mexico; ^6^Coordinación de Tecnología de Alimentos de Origen Vegetal, Centro de Investigación en Alimentación y Desarrollo, A.C. Carretera Gustavo Enrique Astiazarán Rosas, No. 46 Col. La Victoria, 83304 Hermosillo, Sonora, Mexico; ^7^Posgrado en Nanotecnología, Departamento de Física, Universidad de Sonora, Unidad Centro, 83000 Hermosillo, Sonora, Mexico

## Abstract

**Introduction:**

Bacterial agents and oxidative reactions are involved in health and food preservation issues, and *Yucca baccata* (*Y. baccata*) can be a source of compounds with practical applications in both areas, but its investigation remains limited.

**Materials and Methods:**

Butanolic (YBE) and aqueous (YAE) extracts were obtained from the stem of *Y. baccata*. The total saponin, phenolic, and flavonoid contents were analyzed in the YBE and YAE. The antioxidant capacity of the extracts was determined by the DPPH, TEAC, FRAP, and ORAC assays. Seven Gram-positive and five Gram-negative pathogenic bacteria strains were used to determine the MIC and MBC.

**Results:**

Saponin contents were 30% and 1.81% (w/w) in the YBE and YAE, respectively. The total phenolic and flavonoid contents in the extracts were 29.5 *μ*g GAEmg^−1^ (2.95%) and 5.58 *μ*g GAEmg^−1^ (0.56%) in the YBE and 69.92 *μ*g QEmg^−1^ (7.0%) and 1.65 *μ*g QEmg^−1^ (0.165%) in the YAE. The antioxidant capacity values of YBE were 29.18 *μ*g TEmg^−1^, 121.8 *μ*g TEmg^−1^, 33.41 *μ*g TEmg^−1^, and 156.84 *μ*g TEmg^−1^ by the DPPH, TEAC, FRAP, and ORAC assays, respectively. YAE had lower antioxidant values than YBE (*P* < 0.05). Values of 80 mgmL^−1^ and 100 mgmL^−1^ were estimated for MIC and MBC of YBE against the Gram-positive bacteria. Values of 100 mgmL^−1^ and 120 mgmL^−1^ for MIC and MBC of YBE were estimated against the Gram-negative bacteria. No MIC and MBC were obtained for YAE.

**Conclusion:**

YBE exhibited higher antioxidant activity than YAE. Apparently, antibacterial properties of the YBE tended to be higher than those of the YAE.

## 1. Introduction

Bacterial diseases are a major human health issue, especially in children. In 2010, bacterial agents were the leading worldwide cause of foodborne morbidity and mortality, with up to 349 million cases of illnesses and 187 million deaths [1]. These infections are a serious economic burden to the food industry and healthcare sector [2], which worsens with the presence of antibiotic-resistant bacteria. In the U.S., more than 35,000 people die annually from antibiotic-resistant infections, and antibiotic treatments are ineffective [2, 3]. In Mexico, Lemmen et al. [4] found 54 patients with methicillin-resistant Gram-positive bacteria (*Staphylococcus aureus* or enterococci) and 136 with multiresistant Gram-negative bacteria. In 2016, a study in a cancer hospital in Mexico City, from 2004 to 2013, reported *E. coli* (Gram-negative) as the most isolated bacteria (56.5%) from 51,202 urine samples, and 17.3% (*n* = 2,507) showed resistance to betalactamic antibiotics [5]. Similarly, a technical report [6] revealed a high prevalence of resistant strains of *E. coli*, *Staphylococcus aureus*, *Pseudomonas aeruginosa*, and *Klebsiella pneumoniae* (50-100%) recovered from 1,410 biological samples to ampicillin, benzylpenicillin, and sulbactam prescribed by a local public hospital in Northwest Mexico. WHO [7] promoted traditional and complementary/alternative medicine. Natural extracts of plants contain a mix of secondary metabolites (alkaloids, phenols, glycosides, terpenoids, saponins, steroids, flavonoids, tannins, quinones, and coumarins), which have been investigated as new antimicrobial compounds or as a new potential source of antioxidant compounds [8]. On the other hand, it is recognized that antioxidants are beneficial to health because they can prevent cancer, heart diseases, and other chronic degenerative disorders by neutralizing free radicals in body cells [9, 10], and there is a wide variety of rich-antioxidant plants. For example, the methanolic extract of *Peganum harmala* has not only significant free radical scavenging capacity (IC_50_ value of 0.46 ± 0.12 *μ*g mL^−1^) but also total flavonoid and phenol contents (155.29 ± 0.20 mg gallic acid equivalents (GAE) g^−1^ and 39.3 mg of quercetin equivalents g^−1^ of dry matter) [11]. The genus *Yucca* (Family *Asparagaceae*) is particularly found in Central and Northern America [12]. Traditionally, the root of this plant was crushed to obtain a soapy pulp used for personal hygiene and laundry. The fibers of the leaves were woven into sandals, baskets, ropes, and blankets. The tips of the leaves were used as needles for sewing. Its fruits and flowers are still eaten raw, and cooked or mixed with other ingredients. It was also used to treat inflammation of all sorts [13]. *Yucca schidigera* is one of the species extensively investigated on its antimicrobial properties [14]. In addition, *Yucca baccata* (*Y. baccata*), endemic to Northwestern Mexico, seems to have a high concentration of saponins [15]. However, information about its antimicrobial activity or antioxidant capacity remains limited; therefore, the goal of this work was to provide evidence of the antioxidant and antibacterial activity of *Y. baccata*.

## 2. Materials and Methods

### 2.1. Material Collection

*Y. baccata* was harvested in the region of Cananea, Sonora, in Northwestern México during summer, and the collected samples were authenticated by the Herbarium manager Biologist José Jesús Sánchez Escalante using the reference specimen 25075 at the Universidad de Sonora in Hermosillo, Sonora, Mexico.

### 2.2. Reagents Used

n-Butanol was supplied by J.T. Baker (Xalostoc, México) and culture media (Difco®) by Becton Dickinson de México, S.A. de C.V. (CDMX, México). In addition, kanamycin sulphate, diosgenin 98%, and p-anisaldehyde 93% were purchased from Sigma-Aldrich (St. Louis, MO, USA, 2017). Methanol and antioxidant reagents DPPH 2,2-Diphenyl-1-picrylhydrazyl, potassium persulfate, ABTS 2,2′-Azino-bis (3-ethylbenzothiazoline-6-sulfonic acid) diammonium salt, sodium acetate, TPTZ (2,4,6-Tris(2-pyridyl)-s-triazine), hydrochloric acid, fluorescein by Sigma-Aldrich, and 2,2′-Azobis(2-methylpropionamidine) dihydrochloride were purchased to Sigma-Aldrich (Toluca, Mexico). Trolox (6-hydroxy-2,5,7,8-tetramethylchroman-2-carboxylic acid) Millipore was purchased from Merck (México).

### 2.3. Yucca Butanolic and Aqueous Extracts

The *Y. baccata* plant was an adult of 2.23 meters high and a stem of 16 cm diameter. Its stem was cut into slices (2 cm thick and 16 cm in diameter) and left to dry for three weeks at 25–35°C and 19% relative humidity [16]. The dry material was ground (2 mm particle size) using a Thomas-Whiley Model 4 grinder (Laboratory Mill, USA) and stored at 4°C as described by Newbold et al. [17] until analysis. The extract was obtained by soaking the dry material in distilled water (33 g/L) overnight by constantly stirring at 25°C, and the suspension was centrifuged at 1,400 g for 10 min at 4°C. Then the aqueous phase was separated, mixed with an equal volume of n-butanol, and stirred for 30 min at 25°C. The mix was left overnight, and the butanolic phase was separated. This step was repeated three times. The separated aqueous phase was labeled YAE and stored at 4°C. All 3 butanolic phases were mixed, dried (vacuum rotary evaporator Buchi 461, Pace Analytical Services, USA), and filtered (Whatman 1), and the dry powder was labeled YBE and kept at 4°C until analysis [16].

### 2.4. Total Phenolic and Flavonoid Contents

Total phenolic contents in the *Yucca* extracts were determined as follows: sodium carbonate (Na_2_CO_3_) was prepared to 7.5% and a solution of Folin Ciocalteau (1 : 10 v/v); both were diluted in distilled water. Folin's solution was prepared on the same day of analysis. In addition, solutions with different concentrations of gallic acid (from 10 *μ*gmL^−1^ to 500 *μ*gmL^−1^) were prepared to perform the standard curve used to determine the concentrations of *μ*g equivalents of gallic acid (GAE) per milligram of extract. Four replicates of 15 *μ*L of each extract (or standard solution) were added to a microplate well and mixed with 75 *μ*L of Folin's solution and 60 *μ*L of sodium carbonate and left for 30 minutes in darkness. After, the absorbance of each mixture was read at 725 nm in a FLUOstar Omega microplate reader (BMG Labtech Inc., USA) [18]. For the total flavonoid determination, 5% sodium nitrite (NaNO_2_), 10% aluminum chloride (AlCl_3_), and 1 M sodium hydroxide (NaOH) solutions were prepared in distilled water. In addition, different concentrations of quercetin (from 10 *μ*gmL^−1^ to 500 *μ*gmL^−1^) were prepared for the standard curve used to determine the concentrations of *μ*g equivalents of quercetin (QE) per milligram of extract. Three replicates of 100 *μ*L of each extract (YBE and YAE) (or standard solutions) were mixed with 430 *μ*L of sodium nitrite solution and left for 5 minutes. Then, 30 *μ*L of aluminum chloride was added and left to stand for 1 minute; immediately, 440 *μ*L of sodium hydroxide was added and mixed. A 150 *μ*L aliquot of each sample was taken in duplicate, and the absorbance at 496 nm was read on a FLUOstar Omega microplate reader (BMG Labtech Inc., USA) [19].

### 2.5. Antioxidant Capacity

#### 2.5.1. DPPH (1,1-Diphenyl-2-Picrylhydrazyl) Inhibition

First, DPPH radical was prepared on the same day of analysis by weighing 2.5 mg and adding 100 mL of pure methanol; the absorbance of this solution was adjusted to 0.7 ± 0.02 at a wavelength of 515 nm. Simultaneously, different concentrations of Trolox (from 10 *μ*gmL^−1^ to 500 *μ*gmL^−1^) were prepared to make the standard curve used for determining mg of Trolox equivalents (TE) per milligram of extract. Four replicates of 10 *μ*L of each extract (or standard solution) were placed in a microplate well; subsequently, 140 *μ*L of DPPH were added and allowed to stand for 30 minutes in darkness. After, its absorbance at 515 nm was read in less than 1 hour in a FLUOstar Omega microplate reader (BMG Labtech Inc., USA) [19, 20].

#### 2.5.2. TEAC (Trolox Equivalent Antioxidant Capacity)

The ABTS radical was prepared with 19.3 mg of ABTS in 5 mL of distilled water. Simultaneously, 37.8 mg of potassium persulfate (K_2_S_2_O_8_) was added to 1 mL of distilled water, and 88 *μ*L of this mixture was added to the ABTS solution, which was mixed left to stand in the dark for 12-16 h. After, the absorbance of the solution was adjusted to 0.7 ± 0.02 at 754 nm. On the other hand, solutions with different concentrations of Trolox (from 10 *μ*gmL^−1^ to 500 *μ*gmL^−1^) were prepared to build the standard curve used for the determinations of mg of TE per milligram of extract. Four replicates of 2.5 *μ*L of each extract (or standard solution) were mixed with 122 *μ*L of the ABTS solution and left 6 minutes in darkness. Finally, the absorbance of the mixtures at 754 nm was read in a FLUOstar Omega microplate reader (BMG Labtech Inc., USA) [21].

#### 2.5.3. FRAP (Ferric Reducing Antioxidant Power)

Sodium acetate (300 mM, pH 3.6) was prepared (adjusting the pH with concentrated HCl) in distilled water. In a different tube, 10 mM TPTZ (2,4,6-Tris(2-pyridyl)-s-triazine) was prepared in 40 mM HCl. Simultaneously, 20 mM ferric chloride (FeCl_3_) was prepared in distilled water. Finally, 5 mL of sodium acetate, 0.5 mL of TPTZ, and 0.5 mL of ferric chloride were mixed to prepare the FRAP and kept in the darkness. On the other hand, solutions with different concentrations (from 10 *μ*gmL^−1^ to 1000 *μ*gmL^−1^) were prepared to make the standard curve used for determining mg of TE per milligram of extract. Four replicates of 10 *μ*L of each extract and standard solutions were mixed with 140 *μ*L of the FRAP solution and left for 30 minutes in darkness. Finally, the absorbance of the mixtures was read at 590 nm in a FLUOstar Omega microplate reader (BMG Labtech Inc., USA) [21].

#### 2.5.4. ORAC (Oxygen Radical Absorbance Capacity)

The ORAC assay measures the antioxidant effect in the samples expressed as the fluorescence inhibition of fluorescein reagent induced by peroxyl radicals generated by AAPH [2,2′-azobis (2-amidinopropane) dihydrochloride]. The reaction mixture was made up of 150 *μ*L of 10 nM fluorescein, 25 *μ*L of the Trolox standard (standard curve 1.56 - 56 mgmL^−1^), 25 *μ*L of phosphate buffer (75 mM, pH 7.4) as a blank, and 25 *μ*L of each extract. The reaction was started by adding AAPH (240 mM). The reduction in fluorescence was measured every 90 sec by 1.5 h using an excitation wavelength of 485 nm and an emission wavelength of 520 nm in a FLUOstar Omega microplate reader (BMG Labtech Inc., USA). Results were expressed as *μ*g of TE per milligram of each extract, and each reaction was performed in four replicates [22].

### 2.6. ATR-FTIR (Attenuated Total Reflection Fourier Transform Infrared) Analysis and Quantification of Saponins

Saponins were identified in both extracts by ATR-FTIR. The total saponin concentration in the extracts was estimated using a spectrophotometric method [23, 24] based on a specific reaction between the E and F rings of the steroidal sapogenin with p-anisaldehyde and sulfuric acid. Two mg of YBE or YAE were dissolved in 10 mL of methanol. Concentrations of 10, 20, 30, 40, and 50 *μ*g/mL of steroidal sapogenin 93% diosgenin in methanol were prepared to build the standard curve (*R*^2^ = 0.9956) to determine de saponin concentration in the samples. In addition, 0.5% p-anisaldehyde in methanol and 50% sulfuric acid (Sigma-Aldrich) were prepared separately, and 1 mL of each solution was added to the diosgenin preparations homogenized, kept in a water bath for 30 min at 100°C, and left at 25°C. On the other hand, the blank control was a mixture of 2 reagents (1 mL of each) plus 2 mL of methanol. Also, the sample had two mg of YBE or YAE in 10 mL of methanol plus 1 mL of each of the two prepared reagents. A green color produced by the reaction in a quartz cuvette was measured at 430 nm using a Genesys 10s UV-Vis spectrophotometer (Thermo Fisher Scientific Inc., Waltham, MA, USA). Measurements were performed in 3 replicates, and the means were expressed in percentage of YBE or YAE.

### 2.7. Preparation of Bacterial Cultures

The Gram-positive strains including *Staphylococcus aureus* 1789, *Staphylococcus aureus* ATCC 6538, *Staphylococcus aureus* 2072, *Staphylococcus aureus 2997*, *Staphylococcus aureus* 2077, *Staphylococcus aureus* 9062, and *Staphylococcus aureus* 7631 and the Gram-negative strains of *Escherichia coli*, *Escherichia coli* ATCC 25922, *Escherichia coli* O157:H7 ATCC 43890, *Pseudomonas aeruginosa,* and *Salmonella typhi* were used in this study. A loopful of each used strain (0.01 mL) from broth heart infusion (BHI) was cultured separately into 15 mL of Mueller-Hinton broth (MHB) (Difco-Becton, Dickinson and Company, Franklin Lakes, NJ) and incubated at 37°C for 48 h. Then, 0.01 mL of sample from each test tube was cultured separately on Mueller-Hinton agar (MHA) (Difco-Becton, Dickinson and Company, Franklin Lakes, NJ) at 37° C for 24 h.

### 2.8. Determination of Minimum Inhibitory Concentration (MIC) and Minimum Bactericidal Concentration (MBC)

Stock solutions with 250 mgmL^−1^ of YBE and YAE were prepared using 5 mL of BHI broth. The range of tested concentrations were 0, 10, 40, 60, 80, 100, and 120 mgmL^−1^ of YBE and YAE, and they were prepared using BHI broth in agreement to Liu et al. [25]. A negative control was prepared with 295 *μ*L of BHI broth and 5 *μ*L of the bacterial inoculum adjusted to 1.5 × 10^8^ Colony Forming Units/mL^−1^ (CFUmL^−1^). Two positive controls were prepared. One was prepared only with 295 *μ*L of BHI and the other positive control was 1.0 mg of Kanamycin per mL of sterilized distilled water [26]. After, 295 *μ*L of each control and extract concentration was poured into the wells of a microtiter plate. Again, 5 *μ*L of the adjusted inoculum (1.5 × 10^8^ CFUmL^−1^) were added to each well and stirred for 20 seconds. After incubation (37°C for 24 h) and gentle stirring (20 seconds), the reading at 600 nm was recorded in a microplate reader (Fluostar Omega, BMG Labtech, Cary, NC, USA, 2014). Counts of CFU were performed by the drop plate technique [27]. Drops (20 *μ*L) of each serial dilution in triplicate (from 1 × 10^1^ to 1 × 10^7^) and exposed to different YBE or YAE concentrations were poured onto plates of MHA. After incubation (37°C and 24 h), the CFU counts were estimated as follows: CFUmL^−1^ = (mean of the number of colonies/mL cultivates) × dilution factor and expressed as log10 CFUg^−1^ ± standard deviation. Then, the MIC was the lowest concentration of YBE and YAE inhibiting visible growth of bacteria at 37°C for 24 h of incubation. After following a similar protocol, from the test tubes with growth inhibition, 0.01 mL was taken and cultivated on MHA at 37°C for 24 h, and MBC was estimated with the lowest concentration of YBE or YAE which killed 99.9% of the bacteria tested when compared against reference. Each assay for MIC and MBC was performed in duplicate, and the mean of two CFU counts with the standard deviation was recorded. Assays to determine the MIC and MBC was carried out following the recommendations by the Clinical and Laboratory Standards Institute [28].

### 2.9. Statistical Analysis

The variables of CFU counts were log-transformed. Concentrations of saponin, total phenols, flavonoids, and antioxidant capacity were analyzed descriptively. One way ANOVA was used to compare the concentrations of total phenols, flavonoids, and antioxidant capacity between the extracts. The Student *T*-test was used to compare the concentrations of saponin, CFU counts, MIC, and MBC between the extracts. All analyzes were carried out at a significant level of *P* ≤ 0.05 using the STATA 2012 program.

## 3. Results and Discussion

### 3.1. Extraction Yield, Saponin, Phenolic, Flavonoid, and Antioxidant Content in YBE and YAE

The YBE obtained was a fine light brown powder (3.41 g), corresponding to a mean yield of 3.83% (w/w) of dry matter per 89 g of ground *Y. baccata* stem. On the other hand, the YAE was a light brown powder (11.42 g), yielding 12.83% (w/w). Gutierrez-Garcia et al. [16] obtained a mean yield of 7.80% and 24.6% (w/w) of YBE and YAE, respectively, from the *Y. baccata* stem collected in the same geographical region and using the same extraction procedure. It should be noted that *Y. baccata* used by Gutiérrez-García et al. [16] was collected in the spring season, while in this study, samples were collected in the summer season. The extraction yield was associated with the season in which the plant was collected. Similarly, Florez et al. [29] reported a yield of 3.90% from the butanolic extract of *Melissa officinalis* “lemon balm”. Hassan et al. [30] estimated a yield of 4.8 ± 0.6% dry matter of butanolic extracts from Guar meal. However, a higher yields have been reported, e.g., Anamika and Kerketta [31] with 10.09% yield of a butanolic extract of *Y. schidigera* stem, by Krakowska et al. [32] with 14.3% from an ethanolic extract of *Medicago sativa* stem, and Tagousop et al. [33] with 5.5% from a methanolic extract of aerial parts of *Melanthera elliptica*.

On the other hand, in this study, a saponin concentration of 60.07 ± 1.02 *μ*g mL^−1^ was obtained per 200 *μ*g of YBE, which meant a yield of 30%. Conversely, a lower concentration of 3.62 ± 1.32 *μ*gmL^−1^ of saponins was estimated per 200 *μ*g of YAE, which meant a yield of 1.81%. The *Y. baccata* stem had a total saponin concentration of 1.38% (w/w) of dry matter. Tura et al. [34] reported a lower concentration of saponin in the acetone extract of *Phytolacca dodecandra* fruit (7.74%) and *Grewia ferruginea bark* (3.2%) than that in this study. A review by Kregiel et al. [35] pointed out that *Glycyrrhiza glabra* (common name Licorice, root), *Yucca schidigera*, *Quillaja saponaria* (Quillaja bark), *Polygala* spp. (Milkwort), *Primula* spp. (Primula), *Trigonella foenum-graecum* (Fenugreek), *Beta vulgaris* (Sugar beet), and *Hedera helix* (Ivy) were the most known rich-saponins plants with 22.2-32.2%, 10.0%, 9.0-10.0%, 8.0-10.0%, 5.0-10-0%, 4.0-6.0%, 5.8%, and 5.0%, respectively.

On the other hand, the total phenolic contents of YBE and YAE were 29.5 ± 1.16 *μ*g GAE mg^−1^ and 5.58 ± 0.65 *μ*g GAEmg^−1^ of the extracts, representing 2.95% and 0.56% of the extracts' mass, respectively. In addition, the total flavonoid contents of YBE and YAE were 69.92 ± 9.32 mg QEmg^−1^ and 1.65 ± 0.46 mg QEmg^−1^, representing 7% and 0.165% of the extracts' masses, respectively. Both phenolic (*P* < 0.001) and flavonoid contents (*P* < 0.001) were higher in the YBE than in YAE. Zubair et al. [36], reported that *Yucca aloifolia* butanol extract had a phenolic content of 17.4 ± 0.31 mg GAEmg^−1^, which was lower than YBE. On the other hand, ethanolic extracts of the desert plant *Fagonia indica* have a total concentration of phenolics of 4 mg GAE g^−1^ dry plant wt. and flavonoids of 3 g quercetin (Q) % w/dry plant wt. The acetone extracts of *Colotropis procera*, showed a total flavonoid content of 0.9 g Q % w/dry wt., while its ethanolic and acetone extracts contained 4 mg GAE/g dry wt. On the other hand, the acetone extract of *Zygophyllum hamiense* had a flavonoid content of 1.48%, while its ethyl acetate and ethanol extracts had a total phenolic content of 4.0 and 3.61 mg GAE/g dry plant wt., respectively, while the acetone extracts of *Salsola imbricata* had a total of phenolics and flavonoids contents of 4.0 mg GAE/g of plant dry wt. and 3.0 g % w/dry wt., expressed as quercetin, respectively [49]. In contrast, the values of total phenolic and flavonoids previously described are apparently lower than those in the YBE and YAE in this study. In addition, the phenolic and flavonoid content is related to anti-inflammatory, antioxidant, antimicrobial, and cell proliferation activities [35]. These findings may be due to different plant material species, environmental conditions, and extraction protocols performed.

In addition, Hadadi et al. [11] reported that the methanolic extract of the fruit of *Peganum harmala* has a total phenolic and flavonoid contents (39.3 *μ*g of QEmg^−1^ of dry matter) [155.29 ± 0.20 mg GAE g^−1^ dry matters] higher than those found in the YBE. On the other hand, Table 1 shows the antioxidant capacity of YAE and YBE measured by the DPPH, TEAC, FRAP, and ORAC assays. The YBE had higher antioxidant capacity with 23.24, 92.5, 24.63, and 108.12 more units of *μ*g TE/mg than YAE (*P* < 0.05), expressed by the DPPH, TEAC, FRAP, and ORAC methods, respectively. The higher antioxidant activity as observed in the case of butanolic extract (YBE) of *Y. baccata* might be attributed to the better polarity of butanol compared to the aqueous extract (YAE) during the present study [20]. In addition, other *Yucca* species reported different antioxidant potentials, e.g., *Yucca gloriosa* root methanolic extract presented a low TEAC value of 5.78 ± 0.10 *μ*g TEmg^−1^ compared to YBE [18]. Similarly, the butanolic extracts of *Yucca periculosa* showed better antioxidant capacity than its aqueous extract (IC50 2.95 mg and 13.16 mg against DPPH, respectively) [37]. In this context, the antioxidant potential of *Yucca* extracts can be attributed to their phenolic hydroxyl groups, serving as hydrogen donators to near free radicals.

### 3.2. ATR -FTIR

**Figure 1:**
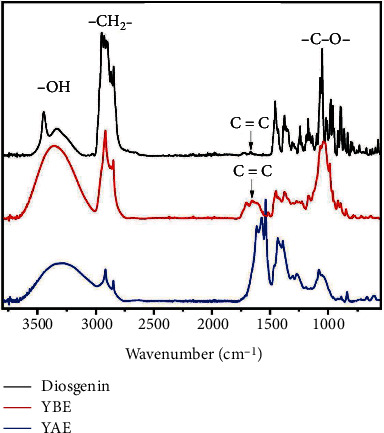
The infrared spectra of the YBE (*Yucca* butanolic extract) and YAE (*Yucca* aqueous extract) using diosgenin as standard.

In Figure 1, the absorption bands centered around 3500-3200 cm^−1^ were assigned to the stretching vibration of -OH groups. Additionally, bands at 2950 cm^−1^, 1750-1700 cm^−1^, 1650-1600 cm^−1^, and 1050 cm^−1^ were assigned to -CH_2_-C=O, -C=C-, and -CO- groups, respectively [38]. The bands shown by the YBE were similar to those generated by the standard. This result provided evidence of the presence of steroidal saponins in the YBE. On the other hand, although smaller, YAE exhibited similar bands to those of the standard (diosgenin) and YBE. This pattern was consistent during the quantification of saponins because YAE showed a lower concentration of steroidal saponins than that contained in the YBE. Furthermore, the YAE extract showed different bands than those observed for the standard or YBE.

### 3.3. The MIC and MBC

Values of 80 mgmL^−1^ and 100 mgmL^−1^ for MIC and MBC of the YBE were estimated against the Gram-positive bacteria tested (Table 2). On the other hand, values of 100 mgmL^−1^ and 120 mgmL^−1^ for MIC and MBC of the YBE were estimated against the Gram-negative bacteria. Apparently, the MIC and MBC values for the Gram-negative bacteria tended to be higher than those of the Gram-positive bacteria (Table 2). It was not possible to determine the MIC and MBC of YAE in this study because the percentages of bacteria inhibition by this extract were always less than 90%.

Kristanti and Punbusayakul [39] reported MIC values of green tea infusions of 50 mgmL^−1^, 75 mgmL^−1^, and 200 mgmL^−1^ for *S. aureus*, *Listeria monocytogenes*, and *E. coli,* respectively, while MIC values for *S. typhimurium* ranged from 100 to 200 mgmL^−1^. The last values were similar to this study and showed that Gram-positive bacteria were more sensitive to green tea infusion than Gram-negative bacteria..Chavasco et al. [40] reported lower MIC values of stem extracts of *Eugenia pyriformis* on *Enterococcus faecalis* (50 mgmL^−1^), *Micrococcus lateus* (50 mgmL^−1^), *S. aureus* (25 mgmL^−1^), *P. aeruginosa* (50 mgmL^−1^), and *S. typhimurium* (50 mgmL^−1^) than those in this study. No MIC value was detected against *E. coli*. It was concluded that Gram-positive bacteria were more sensitive than Gram-negative bacteria to the *Eugenia pyriformis* extracts. Tagousop et al. [33], using butanolic extracts of *Melanthera elliptica,* also found lower MIC values against *E. coli*, *Salmonella flexneri*, and *S. aureus* (256 *μ*gmL^−1^, 128 *μ*gmL^−1^, and 128 *μ*gmL^−1^, respectively) than those in this study. They reported that Gram-positive bacteria were more sensitive than Gram-negative bacteria. Similarly, Elisha et al. [41] studied acetone leaf extracts of some plants (*Hypericum roeperianum*, *Cremaspora triflora*, *Heteromorpha arborescens*, *Pittosporum viridiflorum*, *Bolusanthus speciosus*, *Calpurnia aurea*, *Maesa lanceolate*, *Elaeodendron croceum*, and *Morus mesozygia*). They found that *E. coli* was the most sensitive Gram-negative species (MIC = 0.09 mgmL^−1^), followed by the Gram-negative *P. aeruginosa* (0.12 mgmL^−1^), the Gram-positive *S. aureus* (MIC = 0.20 mgmL^−1^), the Gram-negative *S. typhimurium* (MIC = 0.22 mgmL^−1^), and the Gram-positive *E. faecalis* (MIC = 0.28 mgmL^−1^). However, no difference was found in the average MIC values between the Gram-negative (0.14 mgmL^−1^) and Gram-positive bacteria (0.21 mgmL^−1^), probably due to the similar antibacterial activity exhibited by all plant extracts used against both kinds of bacteria in that study. As described before, based on our MIC and MBC values, the Gram-positive bacteria were apparently more sensitive to the YBE than the YAE in this study. The Gram-positive bacteria have an outer cell wall rich in peptidoglycan, while the Gram-negative bacteria have an outer membrane layer that is external to their poor peptidoglycan cell wall. In addition, lipopolysaccharides and lipoproteins are only present in Gram-negative bacteria [42]. It has been hypothesized that these variations can explain the different sensitivities between the Gram-positive and Gram-negative bacteria to diverse plant extracts. It is proposed that saponins might form cholesterol-saponin complexes and finally lyse cells [43]. However, previous studies have shown that cholesterol is not essential for saponins to provoke disturbs in the membrane [44]. In addition, another study proposed that these differences may be due to the saponin-degradation by glucosidase enzymes produced by the Gram-negative bacteria [45]. However, the cell inhibition mechanism by saponins remains unclear up to now.

On the other hand, though the MIC and MBC for YBE or YAE were relatively high in comparison to others of the aforementioned *in vitro* studies against diverse bacteria, additional investigation testing of the *Y. baccata* extracts *in vivo* are required to know the possible application routes, efficacy, and safety (effectiveness) to determine its real potential usefulness in clinical practice and searching of new drugs.

## 4. Conclusion

There was a good concentration of steroidal saponins in the YBA, but it was poor in YAE. Similarly, the total phenolic, flavonoid contents, and antioxidant capacity were higher in YBE than YAE. Apparently, MIC and MBC values of YBA tended to be higher for Gram-negative bacteria than those for Gram-positive bacteria. The MIC and MBC of YAE could not be estimated. The antioxidant and antimicrobial properties of the butanolic and aqueous *Y. baccata* extracts were assessed for the first time in this study. All obtained findings in this research contribute to the *Y. baccata* bioactive profile showing this plant's pharmaceutical and food relevance.

## Figures and Tables

**Table 1:** Antioxidant capacity values of YAE and YBE were measured by the DPPH, TEAC, FRAP, and ORAC assays.

Extracts	DPPH (*μ*g TEmg^−1^)	TEAC (*μ*g TEmg^−1^)	FRAP (*μ*g TEmg^−1^)	ORAC (*μ*g TEmg^−1^)
YAE	5.94 ± 1.38	29.3 ± 4.4	8.78 ± 0.54	48.72 ± 1.15
YBE	29.18 ± 4	121.8 ± 7.14	33.41 ± 0.11	156.84 ± 5.78

Mean values are the average of four replicated lectures and standard deviation. YAE: *Yucca* Aqueous Extract; YBE: *Yucca* butanolic extract; DPPH: 1,1-diphenyl-2-picrylhydrazyl; TEAC: trolox equivalent antioxidant capacity; FRAP: ferric reducing antioxidant power; ORAC: oxygen radical absorbance capacity.

**Table 2:** Values of MIC and MBC of the YBE for the Gram-positive and Gram-negative bacteria.

Bacteria spp.	MIC (mgmL^−1^)	MBC (mgmL^−1^)
Gram-positive		
*Staphylococcus aureus* 1789	80	100
*Staphylococcus aureus* 2077	80	100
*Staphylococcus aureus* 9062	80	100
*Staphylococcus aureus* 7631	80	100
*Staphylococcus aureus* ATCC 6538	80	100
*Staphylococcus aureus* 2072	80	100
*Staphylococcus aureus* 2997	80	100
Gram-negative		
*Escherichia coli*	100	120
*Escherichia coli* O157 H7	100	120
*Escherichia coli* ATCC 25922	100	120
*Pseudomonas aeruginosa*	100	120
*Salmonella typhi*	100	120

ATCC: American Type Culture Collection; MIC: minimum inhibition concentration; MBC: minimum bactericidal concentration.

## Data Availability

If it is required, the data will be shared.
